# Hepatic Encephalopathy: Early Diagnosis in Pediatric Patients With Cirrhosis

**Published:** 2014

**Authors:** Naghi DARA, Ali-Akbar SAYYARI, Farid IMANZADEH

**Affiliations:** 1. Pediatric Gastroenterology and Hepatology Department, Mofid Children Hospital, Faculty of Medicine, Shahid Beheshti University of Medical Sciences, Tehran, Iran

**Keywords:** Hepatic Encephalopathy, Childhood, Early diagnosis, Cirrhosis

## Abstract

**Objective:**

As acute liver failure (ALF) and chronic liver disease (cirrhosis) continue to increase in prevalence, we will see more cases of hepatic encephalopathy.

Primary care physician are often the first to suspect it, since they are familiar with the patient’s usual physical and mental status. This serious complication typically occurs in patients with severe comorbidities and needs multidisciplinary evaluation and care.

Hepatic encephalopathy should be considered in any patient with acute liver failure and cirrhosis who presents with neuropsychiatric manifestations, decrease level of consciousness (coma), change of personality, intellectual and behavioral deterioration, speech and motor dysfunction.

Every cirrhotic patient may be at risk; potential precipitating factors should be addressed in regular clinic visits. The encephalopathy of liver disease may be prominent, or can be present in subtle forms, such as decline of school performance, emotional outbursts, or depression.

“Subtle form” of hepatic encephalopathy may not be obvious on clinical examination, but can be detected by neurophysiologic and neuropsychiatric testing.

## Introduction

Hepatic encephalopathy refers to a variety of serious but often reversible neurologic abnormalities that arise when the liver cannot detoxify the portal venous blood (Table1) (1).

Brain dysfunction, an important component in the diagnosis of acute liver failure and chronic liver disease, cirrhosis results from an effect of hepatocyte failure on the function of the brain (1-3).

- Management of encephalopathic patients requires immediate identification of precipitating factors and initiation of empiric medical therapy. Current treatments include drugs to prevent ammonia generation in the colon.
- Long-acting benzodiazepines should not be used for treatment of sleep disorders in patients with cirrhosis, because they may precipitate encephalopathy event.

**Table 1:** Classification of hepatic encephalopathy

**Type A**
Encephalopathy associated with acute liver failure
**Type B**
Encephalopathy with portosystemic bypass and no intrinsic hepatocellular disease
**Type C**
Encephalopathy associated with cirrhosis
**Type D**
Encephalopathy associated with disorders of the urea cycle

Therefore, prompt identification and treatment are necessary, since once overt encephalopathy develops the prognosis worsens rapidly.

Thus, pediatric physician and other primary care physicians who care for patients with severe liver disease have an important role in identifying the condition. They will often see the patients when hepetic encephalopathy is in its early stages and its neuropsychiatric manifestations, such as reduced attention, declining or diminishing fine motor skills, or impaired communication are subtle.

The sudden onset and rapid reversibility of encephalopathy in liver disease suggest that it has a metabolic origin, so that the liver cannot detoxify the portal venous blood. The appearance of hepatic ncephalopathy depends on three factors, including portosystemic shunting, alterations in the blood–brain barrier, and the interactions of toxic metabolites with the central nervous system (CNS). Various neurotoxins (especially ammonia) and inflammatory mediators have significant roles in its pathogenesis, inducing low-grade brain edema, and producing a wide range of neuropsychiatric manifestations (4).

Several hypotheses concerning the pathogenesis of hepatic encephalopathy have emerged in recent years, and a number of factors have been reported to directly or indirectly affect brain function in this condition. Ammonia and glutamine are the neurotoxins most often involved in this syndrome (5); others include inflammatory mediators, certain amino acids, and manganese (5,6).

## Portosystemic Shunting

Blood from the intestine can be shunted around the liver via collateral vessels, or in the setting of severe liver disease, through the liver as the blood passes by damaged or necrotic hepatocytes. Potentially neurotoxic nitrogenous intestinal metabolites that normally are removed by the healthy liver, are found in blood circulation in patients with liver dysfunction. Therefore, hepatic encephalopathy is rare if liver function is good. 

Portosystemic shunting may cause encephalopathy, particularly if the patient consumes a high-protein diet (7). If both shunting and hepatocyte dysfunction are present, the patient is most vulnerable for the development of encephalopathy.

## Changes in the Blood–Brain Barrier

The blood–brain barrier plays the important role of isolating the brain from various substances in the systemic circulation. The capillaries of the brain are lined by a specialized endothelium, which is impermeable to a large number of substances. These endothelial cells have no fenestrations otherwise seen in capillaries all over the body. In acute liver failure, the blood–brain barrier goes through changes in permeability, so that marker substances such as inulin, horseradish peroxidase, and trypan blue pass to the brain more readily (8). Therefore, the neurotoxins may directly mediate the changes in blood–brain barrier permeability (9). Although change in the permeability of the blood–brain barrier is seen in the latter stages of encephalopathy, no evidence exists for changes that precede the onset of encephalopathy (10). Zinc, which has a regulatory function in gene transcription and synaptic plasticity, accumulates in the astrocytes, causing relative zinc deficiency and further affecting neurotransmitter synthesis and neurotransmission at the neuronal synapse (6,11).

## Toxic Metabolites

Many potentially toxic substances have been isolated from the blood, CSF, or brain tissue of humans with hepatic encephalopathy. None of these has been shown to be responsible for the mental changes that accompany chronic liver disease and cirrhosis. However, hepatic encephalopathy can occur if toxic nitrogenous substances ingested or formed in the intestine, reach the brain (via a “porous” blood–brain barrier) after incomplete hepatic removal, due to either impaired function of hepatocytes or collateral circulation bypass of the liver (12). The CNS of patients with cirrhosis is hyper-responsive to these circulating toxins (13). 

## Ammonia causes brain swelling

Ammonia is considered an important factor in producing encephalopathy. Ammonia is the byproduct of bacterial metabolism of protein and nitrogenous compounds in the colon and of glutamine metabolism in enterocytes (14). Up to eighty percent is removed from the portal vein blood in its “first pass” from the gut. Hyperammonemia can also be seen in other encephalopathies, such as Reye’s syndrome and organic acidemias. Gut-absorbed ammonia is delivered by the portal vein to the liver, wherein most of it is metabolized into urea, and a small amount is left to be metabolized in the muscles, brain, heart, and kidneys (15). In cirrhosis, less ammonia is metabolized into urea and more of it reaches the astrocytes in the brain. The brain does not have a urea cycle, but metabolizes ammonia to glutamine by glutamine synthase, an enzyme that is unique to astrocytes. Ammonia causes swelling of astrocytes and brain edema by production of glutamine, an osmotically active substance.

## Glutamine causes swelling, oxidative stress

Glutamine draws water into astrocytes and causes changes of type II astrocytosis (also called Alzheimer type II astrocytosis) (5), characterized by swelling, enlarged and pale nuclei, and displacement of chromatin to the periphery of the cell.

Glutamine also facilitate the activation of various receptors, including N-methyl-D-aspartate (NMDA) receptors (15,16), gamma-aminobutyric acid (GABA) receptors, and peripheral-type benzodiazepine receptors on the mitochondrial membrane (17–19). Other inflammatory mediators such as interleukins 1 and 6, tumor necrosis factor, interferons, and neurosteroids can contribute to edema and neurotoxicity (6,17). Increased concentrations of short- and medium-chain fatty acids also are seen in subjects with hepatic encephalopathy. 

High levels are seen in patients with coma; lower levels are associated with lesser degrees of encephalopathy (20).

## Hyponatremia

Hyponatremia (serum sodium concentration<130 mmol/L) is increasingly being recognized as an independent predictor of overt hepatic encephalopathy and is reported to raise the risk by a factor of eight (21).

## Low-grade brain edema

Brain edema occurs in all forms of hepatic encephalopathy, but in cirrhosis it is characteristically of low grade. The mechanism of this low-grade edema has not been known yet. Studies have shown that astrocytes’ swelling is not global, but includes certain parts of the brain and is associated with compensatory extrusion of intracellular myoinositol (22). This, in concomitant with a mild degree of brain atrophy (23) seen in patients with chronic liver disease, is assumed to keep the brain from extreme swelling and herniation, a phenomenon which is usually seen in acute hepatic failure (24,25).

## Transjugular intrahepatic portosystemic shunting and encephalopathy.

The incidence rate of hepatic encephalopathy after placement of a porto-systemic shunt for treatment of portal hypertension ranges from 30% to 55% and is comparable to the rate in cirrhotic patients without a shunt (26) In 5% to 8% of patients, the hepatic encephalopathy is refractory and needs intentional occlusion of the shunt (26,27) Whether to place a portosystemic shunt in a cirrhotic patient, and a history of hepatic encephalopathy depends on the feasible underlying causes of the encephalopathy. For instance, if encephalopathy was precipitated by variceal bleeding, shunt placement will prevent further bleeding and a recurrence of encephalopathy will occur less likely. However, if the encephalopathy is persistent and uncontrollable, shunt placement is contraindicated (27).

- **Clinical manifestations**

The spectrum of symptoms ranges from a subclinical syndrome that may not be clinically obvious (early-stage or “minimal” hepatic encephalopathy) to full-blown neuropsychiatric manifestations, such as cognitive impairment, confusion, slow speech, loss of fine motor skills, peripheral neuropathy, asterixis, clonus, the Babinski sign, decelerate and decorticate posturing, seizures, extrapyramidal symptoms, and coma (4). The clinical manifestations can be reversible with prompt treatment, but recurrence is common, typically induced by an event like infection or gastrointestinal bleeding. 

**Table 2:** Clinical Stages of Hepatic Encephalopathy

	**Clinical Manifestations**	**Asterixis**	**EEG changes**
**Stage I (prodrome)**			
	Slowness of mentation, mild disturbed sleep–awake cycle	Slight	Minimal
**Stage II (impending coma)**			
	Drowsiness, confusion Iinappropriate behavior, disorientation, mood swings	Easily elicited	Usually generalized slowing of rhythm
**Stage III (stupor)**			
	Very sleepy but arousable, Unresponsive to verbal Commands, markedly Confused, delirious, Hyperreflexia, positive Babinski sign	Present if patient cooperative	Grossly abnormal Slowing
**Stage IV (coma)**			
	Unconscious, decerebrate or decorticate, response to pain present (IV A) or absent (IV B)	Usually absent	Appearance of delta waves, decreased amplitudes

- **Minimal hepatic encephalopathy is important to recognize.**

Although this subclinical syndrome is a very early stage, however it is associated with higher rates of morbidity and can affect life quality) 28,29) Abnormal changes in the brain commence at this stage and finally progress to more damage and to the development of overt clinical symptoms. The precise prevalence of minimal hepatic encephalopathy is unknown, because it is difficult to diagnose, but reported rates range from 30% and 84% of patients with cirrhosis (30). The most typical sign of CNS dysfunction is asterixis, a flapping tremor that is demonstrated if the patient’s arms are outstretched and wrists are hyperflexed. There is no tremor at rest but it appears during voluntary movement. Asterixis also is seen in uremia, respiratory failure, and congestive heart failure. Deep tendon reflexes may be exaggerated in early encephalopathy, but in the late stages, the muscles become flaccid and the reflexes disappear. Hyperventilation suggests an extremely poor prognosis.Therefore, minimal hepatic encephalopathy is important to be recognized (28), so that treatment can be initiated.

## Overt encephalopathy and survival

When encephalopathy develops, the prognosis deteriorates rapidly. In patients who do not undergo liver ransplantation, the survival rate at one year is 42%, and the survival rate at three years is 23% in adult groups (31). These rates are worse compared to those after liver transplantation, and the American Association for the Study of Liver Diseases recommends that patients with cirrhosis who develop a first episode of encephalopathy be considered for liver transplantation and be referred to a transplantation center (32).

- **Challenges in diagnosis**

whereas the symptoms of hepatic encephalopathy are not specific and can be subtle in the early stage, its iagnosis can be a challenge.

In subclinical hepatic encephalopathy, the obvious lack of manifestations causes a great diagnostic challenge, but a complete history may reveal personality changes, poor social interaction, and poor performance at work.

Primary care physicians are usually the first to suspect the condition, since they are familiar with the patient’s baseline mental and physical conditions. For instance, the primary care physician may recognize decreased attention and deterioration of memory during a follow up visit, or the physician may ask whether the patient has difficulty in work performance and handwork (psychomotor and fine motor skills). Changes in the electroencephalogram are nonspecific, but electroencephalography can be used for monitoring hepatic encephalopathy (33). At first, a generalized slowing of the pattern and some suppression of the alpha rhythm can be seen. With progression, a highvoltage alpha rhythm emerges with paroxysmal waves of 5 to 7 cycles per second, beginning frontally. 

In deeper coma, there is a generalized slowing, and synchronous low-amplitude 2- to 3-cycle-per-second waves are recorded over the frontal lobes. In spite of the fact that these changes are similar to the progression of hepatic encephalopathy, it is obscure whether routine monitoring of the electroencephalogram provides any advantage over clinical assessment alone. Recorded evoked response potentials can provide some specificity for the diagnosis, but have little clinical use (34).

## Staging the severity of the encephalopathy

A scale for grading clinical encephalopathy is presented in Table 2. This scale is useful for assessing encephalopathy in older patients, but it is of little value in assessing neonates and infants, particularly in the early stages of encephalopathy.

**Table 3:** Precipitating Factors in Hepatic Encephalopathy

Acidosis, alkalosis
Constipation
Diuretic use, dehydration
Gastrointestinal bleeding
Hyponatremia, hypokalemia
Infection
Protein excess
Renal decompensation
Hypoglycemia
Sedative drugs

No study has yet been done on the neuropsychiatric function of children with acute hepatic encephalopathy, and also, an age-dependent grading scale is badly needed. When symptoms are overt, staging should be done to determine the severity of the disease. 

Hepatic encephalopathy may progress rapidly in Acute Liver Failure, with coma developing within hours of the earliest detectable signs. Symptoms often resolve if the precipitating factors are treated (Table 3).

The most common precipitating factors are infections, dehydration, variceal bleeding, and drug toxicity.

## Laboratory tests can identify of metabolic derangements

Although laboratory tests are not diagnostic for hepatic encephalopathy, they can identify metabolic disorders that could contribute to it. Blood ammonia levels are often measured in patients with cirrhosis suspected of having hepatic encephalopathy, but this is not a trustworthy indicator, because many conditions and even prolonged application of tourniquet during blooddrawing can elevate blood ammonia levels (Table 4).

## Imaging can help exclude other diagnoses

Conventional imaging studies of the brain, i.e., computed tomography scan (CT scan) and magnetic resonance image (MRI), are useful only to exclude a stroke, a brain tumor, or an intracranial or subdural hematoma.

- **Several lines of treatment**

Treatment of hepatic encephalopathy involves a preemptive approach to address potential precipitating factors, medical therapy to decrease the production and absorption of ammonia from the gut, and surgical or interventional therapies. 

A multidisciplinary approach for testing the severity of neurologic impairment and response to therapy is required to assist determine if and when liver transplantation is needed.

**Table 4:** Conditions That May Cause Elevated Ammonia Levels

Bacterial overgrowth (may be seen in proton pump inhibitor intake and atrophic gastritis)
Citrullinemia
Drug toxicity (valproic acid)
Extreme exercise
Fulminant hepatic failure
High-protein meals
Inherited disorders of urea cycle
Poor assay technique, e.g., prolonged use of a tourniquet, blood specimen not transported on ice
Portosystemic shunting
Reye’s syndrome
Zinc deficiency

**Prevention of potential precipitating factors**

An important concept in managing hepatic encephalopathy is to recognize that every cirrhotic patient is at risk and to make an effort to address potential precipitating factors in regular clinic visits. This includes review of medication dosing, adverse effects and other toxic substances, and prevention of bleeding from sophageal varices by endoscopic band ligation.

## Diet therapy

The prevalence of malnutrition in cirrhosis may be as high as 100%. All dietary and intravenous protein intake should cease during the acute onset and treatment. Protein may be reintroduced while the encephalopathy subsides. Vitamin and nutritional deficiencies should be assessed by a nutrition specialist, and nutritional needs should be re-evaluated on a regular basis. Protein restriction is no longer recommended and may even be hurtful. Guidelines of the European Society of Parenteral and Enteric Nutrition in 2006 recommended that patients with liver disease should have an energy intake of 35 to 40 kcal/kg of body weight daily, with a total daily protein intake of 1.2 to 1.5 mg/kg of body weight (35). 

Cleansing enemas may decrease further the amount of exogenous ammonia in the intestine, especially if hepatic encephalopathy follows a gastrointestinal hemorrhage. 

Frequent meals and bedtime snacks are helpful to avoid periods of prolonged fasting and muscle protein catabolism and to improve nitrogen balance. Branchedchain amino acids and vegetable protein supplements are recommended to help meet the daily requirements (36). 

## Drug therapy to reduce neurotoxins

Drug treatment is directed at decreasing the neurotoxins which accumulate in cirrhosis. A variety of agents have been used.

**Lactulose **(β-galactosidofructose) is a semisynthetic disaccharide, is a mainstay of treatment for hepatic ncephalopathy (37,38). It has been shown to improve life quality and cognitive function in patients with cirrhosis and minimal hepatic encephalopathy, although it has failed to ameliorate rates of mortality (39). actulose, a cathartic disaccharide, is metabolized into short-chain fatty acids by colonic bacteria. The acidic microenvironment has three major effects:

- It helps the transformation of ammonia to ammonium (NH4+), which is then trapped in the stool, leaving less ammonia to be absorbed;
- It has a cathartic effect;

• It decreases the breakdown of nitrogenous compounds into ammonia (40)

Lactulose has an extremely sweet taste. Its adverse effects include flatulence, abdominal discomfort, and diarrhea. The adult dosage of lactulose is 10–30 mL of the standard syrup (10 g lactulose/15 mL), three times a day. Pediatric dosage is 0.3–0.4 mL/kg, two or three times per day. The dose should be sufficient to acidify the stools (pH less than 6.0) but should not necessarily cause diarrhea.

**Lactitol**, is a nonabsorbable disaccharide that its mode of action is the same as that of lactulose. Its main advantage is that it is produced in a powder form and is thus less sweet and more convenient for use than lactulose. Furthermore, it is not contaminated (as lactulose syrup) with lactose, galactose, and other sugars; therefore, it is more acceptable in patients who are lactose intolerant. It seems to be as efficient as lactulose in the treatment of acute and chronic hepatic encephalopathy and has been found to have a more rapid effect compared to lactose, but with fewer side effects (41).

**Neomycin: **Oral antibiotics have been used successfully in children for the treatment of early hepatic encephalopathy. Tetracycline is used in adults, but is not appropriate for use in children. Neomycin decreases ammonia production, presumably through direct suppression of ammonia forming bacteria (42,43). 

Long term use of neomycin has caused deafness and renal tubular disease (44,45). The adult dosage of neomycin is 4–6 g/d. In children, a starting dose of 1 g is recommended.

**Other antibiotics**, such as metronidazole (Flagyl), neomycin, and vancomycin have been used as alternatives to lactulose, according to the principle that they decrease ammonia producing bacteria in the gut. However, their efficacy in hepatic encephalopathy remains to be determined. In controlled trials, neomycin in combination with sorbitol, magnesium sulfate, or lactulose had the same effectiveness as lactulose, but when used alone, neomycin was no better than placebo (46,47).

**Specific therapy **Management should be in an ICU setting. It is assumed that improved life support, monitoring for the detection of complications, and the management of life-threatening complications in an intensive care setting can increase the overall chance for survival.

The older patient with aggressive delirium is a particular risk to care providers. Restraint and sedation may be required to protect staff. However, sedation usually is not necessary, and benzodiazepines should not be used. Morphine or other opiates may be used in small doses to relieve pain related to monitoring and catheter placement. It is difficult to maintain fluid balance because of the competing forces of a large intake requirement and compromised renal function. Dextrose infusion is indicated to maintain blood glucose concentration. Maintenance fluids consist of 10% dextrose in 0.25 normal saline. 

A total sodium intake of 1.0 mEq/kg/d is usually adequate. Hyponatremia should not be corrected by administration of additional sodium, since the total body sodium overload is the rule. As maintaining a state of hypernatremia may be helpful in the prevention of cerebral edema (48), preventing hyponatremia through maintenance of constant fluid restriction would seem prudent. Potassium requirements may be high, 3–6 mEq/ kg/d, as guided by the serum concentration.

Upper gastrointestinal (GI) bleeding is one of the risk factors that prone the patient to encephalopathy. Thus, anemia should be corrected, maintaining the hemoglobin level above 12 g/dL, to provide maximum oxygen delivery to tissue. Coagulopathy must be managed conservatively; the sometimes massive needs for fresh frozen plasma may cause fluid overload and hypernatremia.

The management of elevated intracranial pressure (ICP) is contingent upon the administration of osmotic substances, usually mannitol. Mannitol is usually administered when alterations in the neurologic examination or the onset of papilledema is detected. 

If intracranial pressure increases more than 30 mmHg, it should also be treated. The doses of mannitol required are usually in the range of 1 g/kg, every 2–6 hours. Serum osmolarity should be monitored during mannitol therapy and should not go beyond 320 mOsm/kg.

**Branched-chain amino acids **(leucine, isoleucine, and valine) (49) have been reported to elevate ammonia intake in muscle and to improve cognitive functions in minimal hepatic encephalopathy (50,51), but they did not reduce the rate of recurrence of hepatic encephalopathy (52). While debate continues over their effectiveness in the management of hepatic encephalopathy, branched -chain amino acids may be used to improve nutritional status and muscle mass of cirrhotic patients. However, the dosing is not standardized, and long-term compliance may be troublesome.

**Other medical therapies **include zinc (53), sodium benzoate (50), and l-ornithine-l-aspartate (54,55), which can stimulate residual urea cycle activities; probiotics (which pose a risk of sepsis from fungi and lactobacilli); and laxatives.

## Liver dialysis

Adsorption of toxins from blood via liver dialysis or use of a non-cell-based liver support system such as MARS (Molecular Adsorbent Recirculating System, Gambro, Inc.) seems to improve the amino acid profile in hepatic encephalopathy, but its role has not been elucidated, and its use is limited to clinical trials (56,57) Transjugular intrahepatic shunts and large portosystemic shunts may need to be closed so as to reverse encephalopathy refractory to drug therapy (26,27,58).

## Liver transplantation

The current scoring system for end-stage liver disease (MELD score) and pediatric end stage liver disease (PELD score) does not include hepatic encephalopathy as a criterion for prioritizing patients on the transplantation list, since it was originally developed to assess shortterm prognosis in patients who undergo transjugular intrahepatic shunting. As a consequence, patients with end-stage liver disease are at increased risk of repeated episodes of encephalopathy, hospital readmission, and death. Hence, the American Association for the Study of Liver Disease suggests referral to a transplantation center when the patient has a first episode of overt hepatic encephalopathy to begin a workup for liver transplantation (32).

Liver transplantation enhances survival in patients with severe hepatic dysfunction, but the presence of neurologic deficits may cause significant morbidity and death (59,60). After transplantation, resolution of cognitive dysfunction, brain edema, and white-matter changes have been reported (58), but neuronal cell death and persistent cognitive impairment after resolution of overt hepatic encephalopathy are also described (61-64).

Whether neurologic impairment will resolve after liver transplantation is related to a number of factors, including the severity of encephalopathy before transplantation; the nature of the neurologic deficits; advanced age; history of alcohol abuse and the existence of alcoholic brain damage; persistence of portosystemic shunts after transplant; emergency transplantation; complications during surgery; and adverse effects of immunosuppressive drugs (59,60,65). 

The optimal timing of liver transplantation is not obviously defined for patients who have had bouts of hepatic encephalopathy, and further study is needed to determine the reversibility of clinical symptoms and brain damage. In these situations, neuropsychiatric testing and advanced neuroimaging can help determine the effectiveness of therapeutic interventions, and it should be considered as a part of the pretransplantation assessment.

## Causes of Death

Cerebral edema with brain death is the direct cause of mortality in most children with ALF. Overwhelming bacterial or fungal infection is seen in a small proportion of patients, and pulmonary failure in very few. 

Hemorrhagic diathesis is a common reason of death in adult patients, but is rare in children afforded effective management. Therapeutic intervention may cause death in some patients. Experience with corticosteroid therapy reveals a risk of duodenal ulcer with perforation or hemorrhage. Extracorporal support devices are associated with technical failure (catheter dislodgment) and with numerous other complications that can lead to death.
